# The Relationship of Cyberchondria, Negative Affect, and Anthropometric Indices with Heart-Rate Variability Among Health Sciences Students: A Psychophysiological Study of Digital Health-Information Behavior

**DOI:** 10.3390/bs16101759

**Published:** 2026-09-28

**Authors:** Tuğçe Orkun Erkılıç, Bülent Bayraktar, Seda Çelikel Taşci

**Affiliations:** 1 Department of Nutrition and Dietetics, Faculty of Health Sciences, Bayburt University, Bayburt 69000, Türkiye; 2 Department of Physiotherapy and Rehabilitation, Faculty of Health Sciences, Bayburt University, Bayburt 69000, Türkiye; bulentbayraktar@bayburt.edu.tr; 3 Department of Nutrition and Dietetics, Faculty of Health Sciences, Erzurum Technical University, Erzurum 25000, Türkiye; seda.tasci@erzurum.edu.tr

**Keywords:** cyberchondria, online health-information seeking, heart-rate variability, cardiac vagal control, screen time, anxiety, digital health behavior

## Abstract

Background: We examined whether cyberchondria was associated with resting heart-rate variability (HRV) in health sciences students. Methods: In this cross-sectional study of 600 students in Türkiye, we assessed cyberchondria using the 12-item Cyberchondria Severity Scale (CSS-12), negative affect using the 21-item Depression Anxiety Stress Scales (DASS-21), self-reported screen exposure, anthropometry, and five-minute resting HRV. The root mean square of successive differences (RMSSD) was the primary physiological outcome. A hierarchical model entered demographics, waist-to-height ratio, and physical activity, then screen exposure and anxiety, followed by CSS-12. Results: CSS-12 correlated inversely with RMSSD (r = −0.412). After covariate adjustment, CSS-12 was associated with lower RMSSD (β = −0.256; *p* < 0.001), adding 4.9% to explained variance (final adjusted R^2^ = 0.272). Waist-to-height ratio showed a smaller inverse association (β = −0.087). Overall, the four-factor CSS-12 model fit was good; comparisons between academic programs were unadjusted and exploratory. Conclusions: Higher CSS-12 scores were associated with lower resting RMSSD after adjustment for measured general anxiety and total screen exposure. Health anxiety was not measured; therefore, the model cannot determine whether this association reflects cyberchondria-related behavior, overlapping health anxiety, or other unmeasured psychological and physiological factors. Prospective studies incorporating validated health-anxiety assessment and detailed digital-behavior and HRV measures are needed.

## 1. Introduction

Seeking health information online is now embedded in everyday self-care. Search engines, social media, symptom checkers, and patient-facing digital platforms can improve access to knowledge and support informed decision-making. Yet the same systems expose users to uncertain, decontextualized, conflicting, and emotionally salient content. For some individuals, searching becomes repetitive and difficult to disengage from, produces escalating concern, and prompts further checking or reassurance seeking. This maladaptive cycle is commonly described as cyberchondria (Starcevic & Berle, 2013; McElroy & Shevlin, 2014; Mestre-Bach & Potenza, 2023). The construct therefore concerns behavior mediated by digital technology rather than technology exposure alone, placing it within the psychological study of human–computer interaction.

Cyberchondria is multidimensional. The Cyberchondria Severity Scale (CSS-12) captures excessiveness, distress, reassurance seeking, and compulsion (McElroy et al., 2019). Recent student-focused scale research likewise supports treating cyberchondria as a multidimensional construct rather than simple search frequency (Hsu, 2024). These dimensions distinguish frequent but purposeful health-information use from a pattern in which search repetition, emotional arousal, interruption of ordinary activities, and demands for reassurance reinforce one another. This distinction matters because raw screen time is an imprecise proxy: the same duration may reflect education, work, social connection, entertainment, or anxious symptom monitoring. A measure focused on these cyberchondria-related features may provide information beyond global digital exposure, although overlap with health anxiety and other forms of distress remains.

Cognitive-behavioral accounts propose that ambiguous bodily sensations trigger threat-focused searches, which increase exposure to rare or severe diagnoses and intensify uncertainty. Temporary reassurance may negatively reinforce renewed searching, while conflicting content can sustain doubt. Consistent with this account, cyberchondria overlaps strongly with health anxiety but retains associations with functional impairment, service utilization, and poorer quality of life (Mathes et al., 2018; Yang & Xu, 2025). A meta-analysis of 20 studies reported a large association between health anxiety and cyberchondria and a smaller association between health anxiety and online health-information seeking, emphasizing that problematic process and emotional consequence—not searching per se—are central (McMullan et al., 2019; Mestre-Bach & Potenza, 2023). Longitudinal evidence further suggests reciprocal relations between health anxiety and online searching (te Poel et al., 2016).

What remains unclear is whether the affective signature of cyberchondria is accompanied by measurable differences in autonomic regulation. Resting heart-rate variability (HRV) indexes beat-to-beat variation arising from interacting cardiac control systems. The root mean square of successive differences (RMSSD) is widely used as a time-domain indicator of vagally mediated HRV, whereas SDNN reflects broader variability over the recording period (Task Force of the European Society of Cardiology and the North American Society of Pacing and Electrophysiology, 1996; Laborde et al., 2017; Agorastos et al., 2023). Higher resting vagally mediated HRV has been theorized to support flexible regulation of attention, emotion, and threat responding; lower HRV has been observed across anxiety-related conditions, although effect sizes and interpretation depend on recording and analytic conditions (Thayer et al., 2012; Chalmers et al., 2014; Agorastos et al., 2023).

A psychophysiological account is especially relevant to cyberchondria. Online symptom searching repeatedly orients attention toward bodily threat, may prolong anticipatory arousal, and can make disengagement difficult. Negative affect could thereby coincide with reduced parasympathetic control. Conversely, lower regulatory capacity may increase perseverative attention and reassurance seeking. Either pathway predicts an inverse cross-sectional relation between cyberchondria and RMSSD, but only temporal or experimental designs can adjudicate direction. The LF/HF ratio is reported here for comparability with the supplied dataset, but it is not treated as a simple measure of “sympathovagal balance,” because that interpretation is physiologically contested (Billman, 2013; Agorastos et al., 2023).

The main analytic question was whether CSS-12 total scores were associated with resting RMSSD after accounting for measured demographics, central adiposity, physical activity, total screen exposure, and general anxiety. Secondary aims were to evaluate overall CSS-12 factor structure and describe correlations and exploratory contrasts across academic programs and CSS-12 score strata. We expected an inverse CSS-12–RMSSD association, while recognizing that unmeasured health anxiety and other determinants prevent a claim of construct-specific physiology.

University students are an especially informative population in which to test these propositions. They are intensive users of search engines, social platforms, and mobile applications; they commonly make independent health decisions for the first time; and they experience academic stress, irregular sleep, and changing access to family and professional reassurance. Health sciences students may be even more exposed to symptom terminology, disease exemplars, and clinical risk information. Their emerging knowledge can improve appraisal, but it can also increase availability of severe diagnoses and encourage self-referential interpretation of benign sensations. Studies of young adults have linked cyberchondria to intolerance of uncertainty, source preference, health anxiety, sleep quality, and eHealth literacy (Zhu et al., 2023). Recent latent-profile evidence further shows that student cyberchondria is heterogeneous, with affect-dominant and reassurance-seeking patterns rather than a single uniform presentation (Yao et al., 2026).

The student context also sharpens the distinction between adaptive and maladaptive online health-information seeking. Searching official guidance for coursework or self-care may be beneficial; persistent cross-checking after reassurance, escalation from common symptoms to catastrophic diagnoses, and interference with study or social activity are qualitatively different. Source credibility, algorithmic ranking, health literacy, and users’ tolerance of uncertainty determine whether information reduces ambiguity or generates additional searching (Sharot & Sunstein, 2020; Schenkel et al., 2021; Bahadır & Dündar, 2024; Zhu et al., 2023). Accordingly, this study treats cyberchondria as a dimensional human–computer interaction pattern and not as a psychiatric diagnosis.

Academic discipline offers a descriptive setting for examining the sample. Program-level differences in exposure to health information could be relevant, but group comparisons require comparable scale functioning and control of demographic and behavioral composition. In the absence of department-specific measurement invariance and adjusted contrasts, any observed program differences are exploratory and cannot be attributed to curricula.

The integration of objective physiology addresses a major gap. Reviews of cyberchondria have been dominated by cross-sectional self-report associations, and a recent scoping review found that all 87 eligible health-impact studies were cross-sectional (Yang & Xu, 2025). Psychophysiological measurement cannot resolve temporal ordering, but it can reduce exclusive reliance on common-method self-report and determine whether CSS-12 scores covary with a theoretically relevant regulatory measure. Examining the CSS-12–RMSSD association after measuring general anxiety and screen time therefore extends questionnaire-only evidence, but it cannot demonstrate an association specific to cyberchondria.

## 2. Materials and Methods

### 2.1. Study Design, Setting, and Participants

A cross-sectional, observational psychophysiological study was conducted at the Faculty of Health Sciences, Bayburt University, Bayburt, Türkiye. Data collection and laboratory physiological assessments took place between March and May 2023. The analytic sample included 600 undergraduate students: 382 women (63.7%) and 218 men (36.3%); 232 Nutrition and Dietetics, 246 Nursing, and 122 Health Management students.

Eligibility required faculty enrollment, age ≥ 18 years, informed consent, and completion of questionnaires and physiological assessment. Reported exclusions were known cardiovascular, neurological, or endocrine disease; treatment affecting autonomic regulation (including beta-blockers, anticholinergics, or psychotropics); active infection or acute illness within 48 h; implanted cardiac devices; and recordings with unresolvable artifacts or ectopic beats >5%. These eligibility exclusions differ from potential residual confounders such as habitual fitness, prior-night sleep, menstrual or contraceptive status, smoking, acute perceived stress, respiration, and adherence to pretest instructions, which were not systematically measured or adjusted.

Of 638 invited students, 618 consented (consent among invitees, 96.9%); 18 were excluded for missing questionnaire data or unusable recordings, leaving 600 analyzed (94.0% of invitees; 97.1% of consenting students). The original report does not identify the recruitment technique, invited population denominator, academic-year composition, or department-specific invitation and participation counts; these should be retrieved from recruitment records if available. A G*Power 3.1.9.7 calculation reported a target of 395 for seven predictors, α = 0.05, power = 0.95, and assumed f^2^ = 0.05; neither the timing nor the empirical basis of this effect-size choice can be verified from the available material, so this calculation should not be described as a prospectively specified requirement.

### 2.2. Ethics and Consent

The study was approved by Bayburt University Ethics Committee (16 December 2022; meeting no. 132.2, decision no. 330) and conducted in accordance with the Declaration of Helsinki. All participants received written information about the purpose, procedures, confidentiality protections, voluntary participation, and right to withdraw, and provided written informed consent before data collection.

### 2.3. Measures

#### 2.3.1. Cyberchondria

Cyberchondria was assessed with the 12-item Cyberchondria Severity Scale (CSS-12; McElroy et al., 2019) in its Turkish adaptation (Tuğtekin & Barut Tuğtekin, 2021). The original validation sources are retained because they document the exact short form and Turkish adaptation used in this study; recent student-focused psychometric research further supports multidimensional assessment of cyberchondria (Hsu, 2024; Yao et al., 2026). Its four three-item dimensions are excessiveness (items 1, 3, and 6), compulsion (2, 7, and 10), distress (4, 8, and 9), and reassurance seeking (5, 11, and 12). Each item uses a five-point scale (1, never; 5, always), giving a total of 12–60; higher scores indicate greater reported cyberchondria. The original study reports permission to use the Turkish version. Overall-sample CFA and reliability results are presented in Section 3.3; equivalence of the measurement model across departments was not assessed.

#### 2.3.2. Negative Affect

Depression, anxiety, and stress symptoms were assessed using the 21-item Depression Anxiety Stress Scales (DASS-21; Lovibond & Lovibond, 1995), adapted into Turkish by Sarıçam (2018). The original and Turkish validation sources are retained because they document the instrument and language version used; a recent study in Turkish university students also used the DASS-21 as a continuous measure of psychological distress (Keskiner et al., 2025). The instrument comprises three seven-item subscales rated on a 4-point Likert scale ranging from 0 (“did not apply to me at all”) to 3 (“applied to me very much or most of the time”). In line with current psychometric recommendations for research contexts, unmultiplied subscale sum scores (potential range: 0–21) were evaluated, with higher scores reflecting greater symptom severity. DASS-21 dimensions were modeled as continuous symptom indicators rather than formal psychiatric categories. The Turkish version demonstrated robust internal reliability in the present sample, with Cronbach’s α values of 0.842 for depression, 0.828 for anxiety, and 0.836 for stress.

#### 2.3.3. Digital Exposure, Physical Activity, and Anthropometrics

Participants estimated their average daily time viewing all digital screens over the previous month using one open-ended recall question, covering smartphones, computers, tablets, and television; this measure does not identify health-information searching. Responses were analyzed continuously and descriptively grouped into 0–2, 3–5, 6–8, and ≥9 h/day. Physical activity was coded as active when reported moderate-intensity activity totaled at least 150 min/week or vigorous-intensity activity at least 75 min/week, otherwise inactive, in relation to WHO adult recommendations. The available materials do not identify the activity instrument, exact item wording, recall period, or detailed scoring procedure; these details require confirmation from original questionnaires. No requirement for three weekly sessions is imposed by the cited WHO volume threshold.

Anthropometric profiling was directly executed by trained researchers following standardized International Society for the Advancement of Kinanthropometry (ISAK) protocols (Esparza-Ros et al., 2019). Body weight (to the nearest 0.1 kg) and body fat percentage (BF%) were measured using a calibrated multi-frequency bioelectrical impedance analyzer (Tanita MC-780, Tanita Corp., Tokyo, Japan) while participants wore light indoor clothing without shoes and had fasted for at least 3 h. Standing height was measured to the nearest 0.1 cm using a wall-mounted stadiometer (Seca 217, Seca GmbH & Co. KG, Hamburg, Germany). Recent method-comparison research in young adults has used the same Tanita MC-780 platform together with standardized anthropometry and a Seca stadiometer, while also emphasizing that BIA-derived fat estimates are not interchangeable with DXA (Mecherques-Carini et al., 2024). Body mass index (BMI) was calculated as weight in kilograms divided by height in meters squared (kg/m^2^). Waist circumference was assessed at the midpoint between the lowest palpable rib margin and the top of the iliac crest at the end of normal expiration using a non-elastic tension-calibrated measuring tape. The waist-to-height ratio (WHtR) was computed as waist circumference divided by height, and the waist-to-hip ratio (WHR) was determined relative to maximal buttocks circumference.

#### 2.3.4. Cardiovascular and HRV Assessment

Blood pressure, mean arterial pressure (MAP), resting heart rate, and HRV were assessed using the reported protocol (Task Force of the European Society of Cardiology and the North American Society of Pacing and Electrophysiology, 1996; Laborde et al., 2017; Agorastos et al., 2023). Measurements took place from 08:30 to 11:30 in a quiet laboratory at 22 ± 1 °C after a 10 min seated acclimatization. Students were instructed to avoid strenuous exercise, caffeine, and alcohol for 24 h and nicotine and heavy meals for at least 3 h before measurement; adherence was not objectively verified. A calibrated Omron M3 Comfort oscillometric device measured duplicate resting blood pressure and heart rate; MAP = diastolic pressure + (systolic pressure − diastolic pressure)/3. The restricted time window reduced variation in measurement time but did not eliminate circadian or individual variation.

Five-minute beat-to-beat (RR) intervals were obtained during quiet upright sitting with spontaneous, unpaced breathing using a Polar H10 chest-strap sensor (Polar Electro Oy, Kempele, Finland). The reported sensor sampling rate was 1000 Hz; this recording should not be described as a clinical multilead ECG. RR-interval series were analyzed in Kubios HRV Premium 3.5.0 (Kubios Oy, Kuopio, Finland), with automatic artifact detection and spline interpolation as described in the study protocol; recordings with persistent artifacts or >5% ectopic beats were excluded. The transfer application/interface and any direct raw-ECG acquisition or independent RR extraction workflow are not specified in the supplied records and must be verified rather than inferred. Published resting-state Polar H10–ECG comparisons support the use of chest-strap RR intervals for selected HRV metrics (Schaffarczyk et al., 2022).

The primary physiological measure was five-minute RMSSD, a time-domain index sensitive to vagally mediated beat-to-beat variability; SDNN and LF/HF were secondary descriptive HRV outcomes (Task Force of the European Society of Cardiology and the North American Society of Pacing and Electrophysiology, 1996; Laborde et al., 2017; Agorastos et al., 2023). For the reported frequency-domain pipeline, artifact-corrected RR intervals were detrended by Smoothness Priors (λ = 500), followed by FFT/Welch spectral estimation; LF (0.04–0.15 Hz) and HF (0.15–0.40 Hz) powers were used to calculate LF/HF. The exact periodogram settings stated in the original protocol (300 s window, 50% overlap) should be checked against the exported Kubios settings because a 300 s window across a 300 s recording may not permit the stated overlap. Respiration was not measured, and LF/HF was not interpreted as a direct sympathetic–parasympathetic balance measure. Contemporary reviews and Polar H10 validation work support explicit reporting of acquisition, artifact correction, recording duration, and spectral-processing settings (Agorastos et al., 2023; Schaffarczyk et al., 2022).

### 2.4. Procedure

Testing sessions were carried out individually between 08:30 and 11:30 AM in a dedicated, sound-attenuated laboratory maintained at a regulated ambient temperature (22 ± 1 °C) and relative humidity (45–55%) to prevent thermal or environmental interference with autonomic tone. Upon arrival, participants submitted written informed consent and underwent initial screening against exclusion criteria.

Assessments were completed in a fixed, standardized sequence: (1) baseline anthropometric evaluation (height, weight, body composition, and waist circumference), (2) a 10 min seated quiet acclimatization period, (3) resting blood pressure and hemodynamic measurements, (4) a 5 min continuous short-term resting HRV acquisition, and (5) self-administered electronic survey completion covering demographic characteristics, lifestyle/physical activity parameters, digital screen exposure, the CSS-12, and the DASS-21. Physiological recordings preceded psychological questionnaire administration to prevent questionnaire-induced cognitive or affective arousal from confounding resting cardiovascular and vagal parameters. All physiological signal files and survey responses were de-identified and linked using a unique numeric study identifier. Raw RR-interval series were inspected immediately following testing; out of the 618 enrolled volunteers, 18 records were excluded due to persistent signal artifacts (>5% ectopic/artifact burden) or incomplete questionnaire items, yielding a final evaluable dataset of *N* = 600.

### 2.5. Statistical Analysis

The reported analyses used SPSS Statistics 28 and AMOS 28 with two-sided α = 0.05. Software versions are reported for reproducibility; analytic validity depends on the specified estimators, diagnostics, and assumptions rather than on describing a software release as the most current. Continuous data were summarized as mean ± SD and categorical data as *n* (%). Complete cases were analyzed after exclusion of 18 incomplete or low-signal-quality records. The original report states that distributional and model diagnostics were visually inspected, but it does not supply residual plots, skewness, heteroscedasticity tests, or a log-RMSSD sensitivity model; no such checks are claimed as newly performed here. Inferences from numerous departmental, correlational, and score-stratum tests are exploratory and use unadjusted *p*-values; no false-discovery-rate correction has been performed.

The four-factor CSS-12 model was estimated with conventional maximum likelihood and summarized using χ^2^/df, CFI, TLI, RMSEA, and SRMR, with factor loadings, composite reliability, and AVE. Because five-point responses are ordinal and neither multigroup invariance nor robust/ordinal-estimator sensitivity results are available, the overall fit does not establish equivalent scores between programs; McDonald’s ω and score median/IQR were not calculable from the available summary results. Pearson correlations describe bivariate associations. Exploratory unadjusted ANOVAs and Tukey comparisons describe academic-program and arbitrary CSS-12 score-stratum contrasts (<30, 30–39, ≥40); these strata are not validated diagnostic thresholds and do not establish a dose–response. The hierarchical linear model used RMSSD as the main outcome: Block 1 entered age, gender, waist-to-height ratio, and physical-activity status; Block 2 entered total daily screen exposure and DASS-21 anxiety; Block 3 added CSS-12 total. This order quantifies the change in model fit when CSS-12 is entered last; it does not establish variance uniquely specific to cyberchondria or causal importance. Anxiety was selected as the most proximal measured affective correlate in the study rationale; depression and stress were not included simultaneously because of conceptual and empirical overlap, and alternative models were not available. Resting heart rate was omitted to preserve the chosen model and because mathematical and physiological coupling complicates adjustment for concurrent heart rate, but an additional heart-rate-adjusted sensitivity model would be informative. No hypothesis or analysis was preregistered.

## 3. Results

### 3.1. Sample Characteristics

The sample comprised 600 undergraduate health sciences students: 232 in Nutrition and Dietetics (38.7%), 246 in Nursing (41.0%), and 122 in Health Management (20.3%) (Table 1). Most participants were women (*n* = 382, 63.7%), and 385 (64.2%) were classified as physically inactive. Mean BMI was 22.86 ± 3.42 kg/m^2^, WHtR was 0.45 ± 0.05, body fat was 23.25 ± 6.18%, and daily screen exposure was 5.24 ± 2.05 h. Mean resting RMSSD was 38.2 ± 11.4 ms, SDNN was 46.8 ± 13.1 ms, heart rate was 74.6 ± 8.6 bpm, MAP was 90.7 ± 8.4 mmHg, and CSS-12 total score was 35.46 ± 7.89.

Screen time was heavily clustered within intermediate and higher exposure brackets: 264 students (44.0%) engaged with screens for 3–5 h/day, 186 (31.0%) for 6–8 h/day, and 72 (12.0%) for ≥9 h/day; in contrast, only 78 students (13.0%) reported low exposure (0–2 h/day). Cumulatively, 258 students (43.0%) had daily screen exposure of 6 h or greater. Mean resting systolic and diastolic blood pressure values were 118.4 ± 10.8 mmHg and 76.8 ± 7.9 mmHg, respectively, with an average LF/HF ratio of 1.82 ± 0.64. Across the DASS-21 dimensions, stress exhibited the highest observed mean (8.16 ± 3.45), followed by anxiety (6.84 ± 3.12) and depression (6.42 ± 3.28). These values provide descriptive physiological and psychological baselines for the cohort without indicating clinical diagnostic status.

### 3.2. Departmental Comparisons

Unadjusted descriptive comparisons indicated small differences in anthropometry between programs (BMI *p* = 0.003; WHtR *p* = 0.002; body fat percentage *p* = 0.004; ηp^2^ = 0.018–0.020). Since neither program-level gender and activity distributions nor adjusted estimates are available in the supplied results, these contrasts should not be interpreted as consequences of program enrollment. Multiple testing was not corrected.

The Nursing group had a higher mean CSS-12 score than the other groups (37.62 ± 8.04 versus 33.84 ± 7.35 and 34.20 ± 7.62; unadjusted F = 16.82, *p* < 0.001, partial η^2^ = 0.053). Other unadjusted program contrasts appear in Table 2. Because department-level scalar invariance and confounder-adjusted comparisons were not tested, these differences cannot establish meaningful between-program latent-mean differences or a curriculum effect and are secondary to the continuous full-sample analyses.

### 3.3. CSS-12 Measurement Model

The hypothesized four-factor CSS-12 model showed strong global fit, chi-square(48) = 108.42, *p* < 0.001, chi-square/df = 2.26, CFI = 0.981, TLI = 0.974, RMSEA = 0.046, 90% CI [0.034, 0.058], and SRMR = 0.036. All standardized loadings were substantial (0.732–0.842) and statistically significant where freely estimated. Composite reliability ranged from 0.811 to 0.865 and AVE from 0.589 to 0.681, supporting internal consistency and convergent validity (Table 3). Overall model fit does not demonstrate configural, metric, or scalar invariance across academic departments; group mean interpretations are therefore restricted.

At the factor level, reassurance seeking showed the lowest loading range (0.732–0.810), whereas distress included the largest loading (0.842). Nevertheless, each factor exceeded conventional CR and AVE benchmarks. Distress had the highest α (0.862), CR (0.865), and AVE (0.681); reassurance seeking had the lowest corresponding estimates (α = 0.805, CR = 0.811, AVE = 0.589). The results indicate that all four domains were measured adequately while preserving meaningful differentiation among excessive searching, emotional distress, reassurance behavior, and interference/compulsion.

### 3.4. Bivariate Associations

Cyberchondria was moderately associated with daily screen time (r = 0.435), anxiety (r = 0.512), and stress (r = 0.486). It was also positively associated with resting heart rate (r = 0.318), MAP (r = 0.246), and LF/HF ratio (r = 0.394), and inversely associated with RMSSD (r = −0.412), all *p* < 0.001. RMSSD showed its largest bivariate inverse correlations with resting heart rate (r = −0.498), anxiety (r = −0.468), and stress (r = −0.432). The correlations support shared variance among cyberchondria, negative affect, and cardiac autonomic function but do not establish a directional pathway (Table 4).

Daily screen time correlated positively with anxiety (r = 0.348), stress (r = 0.362), resting heart rate (r = 0.284), MAP (r = 0.198), and LF/HF ratio (r = 0.310), and inversely with RMSSD (r = −0.326). WHtR showed smaller associations with cyberchondria (r = 0.184), RMSSD (r = −0.216), and MAP (r = 0.294). Anxiety and stress were strongly intercorrelated (r = 0.648), but neither this correlation nor the later VIF estimates suggested statistical redundancy. RMSSD and LF/HF ratio were inversely related (r = −0.614), providing convergent but not physiologically interchangeable information.

### 3.5. Exploratory Cyberchondria Score-Stratum Comparisons

In a secondary descriptive analysis using arbitrary, nonclinical score bands, mean RMSSD was 44.8 ± 10.2 ms in the lowest band and 31.2 ± 9.1 ms in the highest (unadjusted F = 58.42, *p* < 0.001). Other unadjusted outcomes appear in Table 5. Selection of cutpoints from the CSS-12 distribution can create an apparent ordered pattern; the continuous association and adjusted model are the principal analyses.

For transparency, Table 5 reports the full descriptive results for the exploratory bands. These contrasts are not independent confirmation of a dose–response relation, and their *p*-values have not been corrected for multiplicity.

### 3.6. Hierarchical Regression of RMSSD

As shown in Table 6, in the reported hierarchical model, Block 1 accounted for R^2^ = 0.091; adding screen exposure and anxiety increased R^2^ by 0.141 to 0.232; CSS-12 entered last, adding ΔR^2^ = 0.049 (final R^2^ = 0.281; adjusted R^2^ = 0.272). CSS-12 was inversely associated with RMSSD (B = −0.37, SE = 0.06, β = −0.256, 95% CI [−0.49, −0.25], *p* < 0.001). These estimates are conditional on the selected block order and measured covariates. Neither health anxiety nor several physiological and behavioral determinants were adjusted; the outcome distribution and residual diagnostic plots cannot be independently audited from the summary table.

WHtR also showed a relatively small standardized inverse association (β = −0.087), and physical inactivity was associated with lower RMSSD. Correlations among CSS-12, general anxiety, screen exposure, and resting heart rate (including HR–RMSSD r = −0.498) make attribution to cyberchondria alone inappropriate. VIF diagnostics address numerical collinearity but do not remove conceptual overlap or confounding.

## 4. Discussion

### 4.1. Principal Findings and Contribution

The central observation is a moderate inverse CSS-12–RMSSD association that persists in the specified regression after adjustment for measured demographic, anthropometric, activity, screen-time, and general anxiety variables (ΔR^2^ = 0.049). The four-factor CSS-12 model showed good fit overall. Department and score-band analyses are exploratory: invariance, covariate adjustment, and multiplicity control were not established. Because health anxiety and several physiological determinants were not measured, the association cannot be interpreted as specific to cyberchondria. The cross-sectional design also does not indicate whether digital behavior preceded autonomic variation.

The study’s primary theoretical contribution is to connect cyberchondria with neurovisceral models of regulatory flexibility. RMSSD reflects vagal modulation of the heart under specified recording conditions and is often interpreted as a peripheral marker relevant to flexible emotional and attentional regulation (Thayer et al., 2012; Laborde et al., 2017; Agorastos et al., 2023). The observed association does not mean that cyberchondria “suppresses” vagal tone or causes autonomic dysfunction. A more defensible interpretation is that higher cyberchondria scores identify participants who, at rest, also show lower vagally mediated HRV. This psychophysiological covariance is compatible with—but does not prove—a threat-monitoring and perseveration mechanism.

### 4.2. Exploratory Program-Level Observations

Health-professions education changes both exposure to medical information and the contexts in which bodily symptoms are interpreted. However, the program comparisons in this study were secondary, unadjusted, and not supported by department-level measurement-invariance testing; they are therefore treated only as descriptive context.

Observed differences in CSS-12 scores, negative affect, anthropometry, and HRV may reflect student composition, self-selection, year of study, clinical exposure, lifestyle, or other unmeasured factors. They should not be interpreted as effects of program enrollment or curricula.

The higher cyberchondria and anxiety profile observed among Nursing students is compatible with evidence that cyberchondria co-occurs with anxiety and problematic Internet use in nursing-student samples (Varer Akpinar et al., 2023). This contextual consistency does not establish a discipline effect, because the present contrast was unadjusted and health anxiety was not measured.

Other studies emphasize heterogeneity within student populations rather than simple discipline effects, including profiles dominated by distress, reassurance seeking, or problematic searching (Yao et al., 2026). Accordingly, individual-level and full-sample analyses remain the primary basis for interpretation, while program-level findings warrant adjusted, multisite replication.

### 4.3. Cyberchondria, Screen Exposure, and Negative Affect

Cyberchondria is expected to correlate with anxiety because ambiguous somatic information activates threat appraisal, searching increases exposure to severe diagnostic possibilities, and reassurance frequently decays. Intolerance of uncertainty, anxiety sensitivity, metacognitive beliefs, and compulsive checking can maintain this loop. Recent synthesis confirms that anxiety is the most frequently reported psychological correlate, while depression, obsessive-compulsive symptoms, sleep disturbance, and impaired daily functioning are also represented (Schenkel et al., 2021; Yang & Xu, 2025).

Cyberchondria and DASS-21 anxiety were correlated (r = 0.512), and CSS-12 added explained RMSSD variance when entered after DASS-21 anxiety. However, DASS-21 measures general anxiety symptoms, not illness-specific health anxiety; the latter is closely connected with repeated symptom searching and was not measured. The regression therefore cannot isolate a cyberchondria association independent of health anxiety. McMullan et al. (2019), Mestre-Bach and Potenza (2023), Yang and Xu (2025), and the 2026 Tunisian student report of Chaari et al. likewise situate health anxiety alongside cyberchondria, although none provides a direct test of this study’s HRV association.

Prior work documents the close relationship of cyberchondria with anxiety and uncertainty (McMullan et al., 2019; Schenkel et al., 2021; Arsenakis et al., 2021). A 2026 student profile study (Yao et al., 2026) reports heterogeneity in affective and searching patterns, whereas a 2026 Tunisian student cross-sectional report (Chaari et al., 2026) underscores health-anxiety co-occurrence. Neither comparison establishes that cyberchondria independently affects HRV; differences in design, population, and health-anxiety measurement constrain direct comparison.

Not every student study reports high cyberchondria or consistent demographic differences. Some cohorts show low-to-moderate mean levels, no gender difference, or weaker associations with eHealth literacy and source use (Bahadır & Dündar, 2024; Yao et al., 2026). Differences may arise from the CSS version used, total-score scaling, pandemic versus post-pandemic timing, inclusion of students who never search for health information, medical/health sciences composition, local healthcare access, and whether eHealth literacy is self-perceived or objectively demonstrated. Health sciences students may search more competently yet encounter more disease content; knowledge may therefore act as either a protective appraisal resource or an exposure amplifier.

Related evidence from nursing and general student samples further illustrates contextual variability. Varer Akpinar et al. (2023) linked cyberchondria with COVID-19 anxiety and Internet addiction among nursing students, whereas broader studies show that search motives and source selection can be as important as exposure duration (Lagoe & Atkin, 2015; Bahadır & Dündar, 2024). Pandemic-era increases in disease salience and uncertainty also intensified health-related searching in some populations (Vismara et al., 2021; Mestre-Bach & Potenza, 2023). Consequently, differences between studies may reflect historical context and task-specific digital behavior rather than failure of the underlying model.

Screen time was also related to cyberchondria, yet its correlation was smaller than that of anxiety, and its standardized association with RMSSD attenuated after CSS-12 scores were entered. Time online is an exposure quantity, whereas CSS-12 scores summarize reported repetition, distress, interference, and reassurance seeking during online health-information behavior. Previous and current syntheses similarly emphasize that online health searching ranges from adaptive information acquisition to escalating threat-focused checking (Muse et al., 2012; Baumgartner & Hartmann, 2011; Singh & Brown, 2014; Mestre-Bach & Potenza, 2023; Yang & Xu, 2025). These findings support measuring the content and function of searching in addition to duration, but do not establish that the RMSSD association is unique to cyberchondria.

The moderate association with stress likewise fits an arousal-based interpretation. Repetitive searches may sustain cognitive load through conflicting advice, rare-disease salience, and repeated self-monitoring. Conversely, individuals already experiencing stress may search more often or interpret neutral information as threatening. The cross-sectional design cannot distinguish these possibilities. The reciprocal relation reported longitudinally between health anxiety and online health-information seeking (te Poel et al., 2016) supports feedback-loop models in which predisposition and digital behavior reinforce one another over time.

### 4.4. Autonomic Profile and Possible Mechanisms

Resting HRV reflects dynamic modulation of cardiac timing by central and peripheral regulatory systems. RMSSD is predominantly sensitive to short-term vagally mediated variation, while SDNN captures broader variability over the measurement window. Reduced HRV is reported across stress-related and psychiatric conditions, but values depend strongly on posture, respiration, recording length, physical fitness, medication, sleep, and signal processing (Task Force of the European Society of Cardiology and the North American Society of Pacing and Electrophysiology, 1996; Laborde et al., 2017; Agorastos et al., 2023).

The inverse CSS-12–RMSSD association agrees in direction with broader reports relating anxiety and stress to reduced resting HRV (Chalmers et al., 2014; Agorastos et al., 2023). Yet the present study did not assess search episodes, experimentally manipulate information exposure, or adjust for health anxiety. Its ΔR^2^ of 0.049 describes incremental model fit given the block order and should not be interpreted as an effect on vagal physiology.

Although direct HRV studies of cyberchondria remain scarce, the present direction converges with the wider anxiety and perseverative-cognition literature. Chalmers et al. (2014) reported reduced HRV across anxiety disorders; Thayer et al. (2012) linked HRV to neural systems involved in threat appraisal and regulation; and Agorastos et al. (2023) summarized increased heart rate and reduced HRV across psychiatric and stress-related states. The ordered decline in RMSSD and SDNN alongside increasing anxiety is therefore biologically coherent with an arousal-regulation framework.

Some studies find small, heterogeneous, or context-dependent relations between subjective distress and resting HRV, and HRV cannot be interpreted as a direct measure of a single psychological process. Null or weaker findings may reflect brief recordings, uncontrolled breathing, nonstationary data, different age/fitness distributions, medication, or use of frequency-domain ratios as primary outcomes. The comparatively clear pattern here may partly reflect the large *N* = 600, the restricted young-adult context, and simultaneous modeling of screen exposure and anxiety. Conversely, if recording conditions were insufficiently standardized, the association could be inflated or attenuated by unmeasured behavioral and physiological factors; complete protocol reporting is therefore decisive.

Several explanations remain compatible with the observations: repeated health searching could accompany sustained arousal; lower resting regulatory flexibility could precede persistent checking; or shared determinants including health anxiety, sleep, respiratory pattern, smoking, fitness and acute stress could influence both. Longitudinal or within-person designs measuring search behavior and respiration are needed to distinguish these possibilities.

The positive LF/HF findings require caution. Although the ratio increased with CSS-12 scores and correlated inversely with RMSSD, LF/HF is not a direct or unitary index of sympathetic–parasympathetic balance (Billman, 2013; Agorastos et al., 2023). Its value here is descriptive and convergent, not mechanistic. RMSSD is the more interpretable primary vagal index, provided that recording duration, respiration, posture, artifacts, and recent behavior were appropriately controlled and reported.

### 4.5. Measurement Implications for Human–Computer Behavior Research

Cyberchondria is not synonymous with frequent Internet use. Its measurement requires attention to repeated searching, distress, interference, and reassurance seeking. Reviews have warned that the field relies heavily on variants of the CSS and that inconsistent factor structures or arbitrary thresholds can hinder comparison (McElroy et al., 2019; Schenkel et al., 2021; Mestre-Bach & Potenza, 2023; Hsu, 2024; Yang & Xu, 2025).

The CFA findings support retaining the multidimensional CSS-12 structure. Excessiveness describes repeated searching, distress captures adverse emotional impact, reassurance seeking reflects behavioral escalation toward professional confirmation, and compulsion captures interference with daily activity. These processes may not have identical physiological correlates. The current tables support the measurement model but do not provide factor-specific HRV regressions. Future work should test whether distress and compulsion are more closely linked to autonomic measures than excessiveness, and whether reassurance seeking acts as a short-term regulator that maintains the longer-term cycle.

The four-factor solution agrees with the CSS-12 development study (McElroy et al., 2019) and with recent student work using the same dimensional structure (Yao et al., 2026). In contrast, studies using the original 33-item CSS, translated forms, or profile analysis sometimes produce alternative organization or heterogeneous subgroups. These differences do not necessarily invalidate the construct: they may indicate that affective distress and overt searching/reassurance behavior combine differently across cultures, educational backgrounds, and digital environments. Testing measurement invariance by gender, program, and language would be more informative than assuming that identical total scores reflect identical behavioral profiles.

Measurement quality also extends beyond the cyberchondria scale itself. National surveys use heterogeneous items for online health-information seeking, limiting comparability of frequency, source, motivation, and consequence measures (Choi & Jeong, 2021). Future studies should pair CSS-12 dimensions with objective browser or smartphone logs, validated health-anxiety measures, source credibility, and functional outcomes. Evidence that health anxiety can mediate relations between somatic symptoms and cyberchondria (Santoro et al., 2022) and that social support may buffer health-anxiety-related quality-of-life impairment (Ciułkowicz et al., 2022) identifies additional variables for an expanded model.

The results also caution against clinical labels based solely on score bands. The low, moderate, and high groups in this manuscript are exploratory CSS-12 score strata, not diagnostic categories. Ordered differences are useful for displaying pattern and effect size, but dimensional analyses should remain primary. Validation against impairment, clinical interview, longitudinal persistence, and behavioral search logs would be needed before proposing clinically meaningful thresholds.

### 4.6. Practical Implications

These associations motivate prospective studies of online health-search behavior and source appraisal in student health settings. The present design did not test an intervention, misinformation exposure, or clinical benefit, and cannot support recommendations that any specific digital-literacy intervention would change RMSSD.

For health sciences education, the findings suggest that factual health knowledge should be paired with digital source appraisal and uncertainty management. Students need to distinguish educational searching from self-diagnostic escalation, understand how ranking algorithms and anecdotal salience shape perceived probability, and know when further searching is unlikely to add clinical value. Qualitative evidence shows that young people face practical difficulties appraising online health information, while surveys demonstrate substantial variation in digital health literacy and search behavior (Chu et al., 2017; Dadaczynski et al., 2021; Hsu et al., 2021; Zhu et al., 2023; Bahadır & Dündar, 2024; Hsu, 2024). Training should therefore evaluate applied appraisal skills rather than assume that frequent Internet use or self-rated eHealth literacy equals competence.

The findings may also matter for student health services. Reassurance seeking can increase consultations, yet dismissing online concerns may damage trust. Systematic evidence indicates that online health information can both support and strain clinician–patient relationships (Luo et al., 2022). A constructive response is to ask what was searched, identify the feared interpretation, review source credibility, and establish a bounded plan for monitoring symptoms. Such an approach addresses the searching cycle without stigmatizing students or discouraging appropriate care.

### 4.7. Strengths, Limitations, and Future Directions

Strengths include the large sample, simultaneous assessment of digital behavior, affect, cardiovascular measures, and HRV, explicit evaluation of the CSS-12 measurement model, and hierarchical modeling that places CSS-12 scores alongside screen duration and measured general anxiety. Reporting effect sizes and change in explained variance improves interpretability, although neither feature establishes construct specificity.

Limitations and possible directions of bias require explicit consideration. Cross-sectional observation leaves temporal order unresolved and reverse causation possible (Yang & Xu, 2025). Unmeasured health anxiety may inflate the apparent CSS-12 association beyond adjustment for general anxiety (McMullan et al., 2019; Chaari et al., 2026). One-question self-reported screen exposure can misclassify both duration and content; it cannot identify health searches. Physical activity dichotomization and unknown question wording may leave residual fitness-related confounding. Time of day, posture, and temperature were standardized by protocol, whereas respiration, prior-night sleep, menstrual/contraceptive factors, smoking, and verified pretest adherence were not measured; these omissions can alter resting HRV independently of cyberchondria (Laborde et al., 2017; Agorastos et al., 2023). Chest-strap RR intervals are distinct from verified raw ECG, and the acquisition interface remains unspecified (Schaffarczyk et al., 2022). Neither department-level measurement invariance nor adjusted program comparisons are available, so apparent program differences can reflect measurement bias or student composition. Uncorrected multiplicity increases false-positive risk among exploratory outcomes; arbitrary CSS-12 score bands can suggest an artificial dose gradient. The one-university sample and unknown eligible-population and program-specific participation denominators limit generalizability. Finally, original residual diagnostic outputs and alternative models (log RMSSD, resting HR, stress/depression) are unavailable, limiting confidence in model robustness.

Future studies should combine device-recorded search behavior with ecological momentary assessment and ambulatory HRV, allowing within-person tests before, during, and after health searches. Longitudinal cross-lagged and random-intercept models could separate stable vulnerability from temporal coupling. Experiments could compare neutral information seeking with uncertain or threat-escalating search tasks while controlling respiration. Finally, preregistered mediation models should test intolerance of uncertainty, health anxiety, sleep disruption, and perseverative cognition as candidate pathways.

## 5. Conclusions

We aimed to examine whether CSS-12 scores were inversely associated with resting RMSSD after adjustment for measured covariates. The observed inverse correlation and change in model R^2^ support that statistical association in this sample. They do not establish causal direction, an effect specific to cyberchondria, independence from unmeasured health anxiety, or a clinically meaningful autonomic deficit. Overall CSS-12 factor fit was good, whereas program-level and score-stratum contrasts remain exploratory. Replication with measured health anxiety, validated activity and search-exposure instruments, full HRV acquisition details, and longitudinal data is needed before practical or mechanistic conclusions can be drawn.

## Figures and Tables

**Table 1:** Demographic, departmental, anthropometric, affective, and autonomic characteristics (*N* = 600).

Characteristic/Domain	Sub-Category/Measure	*n*	%	Mean ± SD
Academic Department	Nutrition and Dietetics	232	38.7	—
	Nursing	246	41.0	—
	Health Management	122	20.3	—
Gender	Women	382	63.7	—
	Men	218	36.3	—
Regular Physical Activity	Inactive/Sedentary	385	64.2	—
	Active/Regular exercise	215	35.8	—
Daily Screen Exposure	0–2 h	78	13.0	—
	3–5 h	264	44.0	—
	6–8 h	186	31.0	—
	≥9 h	72	12.0	—
	Continuous exposure (hours/day)	600	100	5.24 ± 2.05
Anthropometry & Adiposity	Body weight (kg)	600	100	63.84 ± 11.20
	Height (cm)	600	100	167.1 ± 8.54
	BMI (kg/m^2^)	600	100	22.86 ± 3.42
	Waist circumference (cm)	600	100	76.52 ± 8.76
	Waist-to-height ratio (WHtR)	600	100	0.45 ± 0.05
	Waist-to-hip ratio (WHR)	600	100	0.78 ± 0.07
	Body fat percentage (BF%)	600	100	23.25 ± 6.18
Hemodynamics	Systolic blood pressure (mmHg)	600	100	118.4 ± 10.8
	Diastolic blood pressure (mmHg)	600	100	76.8 ± 7.9
	Mean arterial pressure (MAP, mmHg)	600	100	90.7 ± 8.4
	Resting heart rate (bpm)	600	100	74.6 ± 8.6
Cardiac Autonomic Indices (HRV)	RMSSD (ms)	600	100	38.2 ± 11.4
	SDNN (ms)	600	100	46.8 ± 13.1
	LF/HF ratio	600	100	1.82 ± 0.64
Affective Symptoms (DASS-21)	Anxiety	600	100	6.84 ± 3.12
	Stress	600	100	8.16 ± 3.45
	Depression	600	100	6.42 ± 3.28
Cyberchondria (CSS-12)	Total score	600	100	35.46 ± 7.89

Note. BF% = body fat percentage; BMI = body mass index; CSS-12 = 12-item Cyberchondria Severity Scale; DASS-21 = Depression Anxiety Stress Scales-21; HRV = heart-rate variability; MAP = mean arterial pressure; RMSSD = root mean square of successive differences; SDNN = standard deviation of normal-to-normal intervals; LF/HF ratio = Low-frequency to high-frequency ratio; WHR = waist-to-hip ratio; WHtR = waist-to-height ratio; — = not applicable.

**Table 2:** Comparison of anthropometric, cyberchondria, affective, and autonomic parameters across academic departments (*N* = 600).

Variable	Nutrition & Dietetics (*n* = 232)	Nursing (*n* = 246)	Health Management (*n* = 122)	F	*p*	ηp^2^	Post Hoc (Tukey HSD)
Age (years)	20.82 ± 1.64	21.04 ± 1.72	20.91 ± 1.68	1.18	0.308	0.004	—
BMI (kg/m^2^)	22.31 ± 3.15	23.12 ± 3.52	23.38 ± 3.58	5.84	0.003	0.019	Nutr < Nurs, HM
WHtR	0.44 ± 0.05	0.46 ± 0.06	0.46 ± 0.05	6.12	0.002	0.020	Nutr < Nurs, HM
BF%	22.14 ± 5.92	23.98 ± 6.24	23.86 ± 6.30	5.46	0.004	0.018	Nutr < Nurs, HM
CSS-12 Total	33.84 ± 7.35	37.62 ± 8.04	34.20 ± 7.62	16.82	<0.001	0.053	Nurs > Nutr, HM
Excessiveness	8.72 ± 2.14	9.68 ± 2.38	8.86 ± 2.22	11.45	<0.001	0.037	Nurs > Nutr, HM
Distress	8.45 ± 2.26	9.54 ± 2.51	8.58 ± 2.34	14.28	<0.001	0.046	Nurs > Nutr, HM
Reassurance Seeking	7.98 ± 2.18	9.02 ± 2.42	8.14 ± 2.26	13.91	<0.001	0.045	Nurs > Nutr, HM
Compulsion	8.69 ± 2.10	9.38 ± 2.32	8.62 ± 2.20	7.64	0.001	0.025	Nurs > Nutr, HM
DASS-21 Anxiety	6.42 ± 2.94	7.58 ± 3.24	6.14 ± 2.88	11.96	<0.001	0.039	Nurs > Nutr, HM
RMSSD (ms)	39.84 ± 11.12	36.14 ± 11.38	39.22 ± 11.40	7.15	0.001	0.023	Nurs < Nutr, HM
LF/HF ratio	1.72 ± 0.58	1.94 ± 0.69	1.76 ± 0.61	6.84	0.001	0.022	Nurs > Nutr, HM

Note. Values are mean ± SD. One-way ANOVA with Tukey HSD post hoc comparisons. BF% = body fat percentage; BMI = body mass index; CSS-12 = 12-item Cyberchondria Severity Scale; DASS-21 = Depression Anxiety Stress Scales-21; HM = Health Management; Nurs = Nursing; Nutr = Nutrition and Dietetics; RMSSD = root mean square of successive differences; WHtR = waist-to-height ratio; LF/HF ratio = Low-frequency to high-frequency ratio; — = not applicable.

**Table 3:** Confirmatory factor analysis and reliability of the CSS-12 (*N* = 600).

Factor	Item	Item Content (Abbreviated)	λ	SE	z	*p*	α	CR	AVE
Excessiveness	SIBER1	Online query for vague somatic signs	0.772	—	—	—	0.834	0.836	0.630
	SIBER3	Cross-checking multiple medical websites	0.798	0.054	19.30	<0.001			
	SIBER6	Repetitive search patterns	0.810	0.053	20.21	<0.001			
Distress	SIBER4	Panic reaction to differential diagnoses	0.794	—	—	—	0.862	0.865	0.681
	SIBER8	Perceived health state aggravation	0.842	0.051	21.27	<0.001			
	SIBER9	Post-search affective arousal and strain	0.839	0.050	22.04	<0.001			
Reassurance Seeking	SIBER5	Triggers clinical consultation	0.732	—	—	—	0.805	0.811	0.589
	SIBER11	Proposing complex laboratory workups	0.758	0.062	17.18	<0.001			
	SIBER12	Demanding secondary specialist opinion	0.810	0.059	19.39	<0.001			
Compulsion	SIBER2	Disruption of routine cognitive/web tasks	0.789	—	—	—	0.848	0.851	0.656
	SIBER7	Interference with occupational/study efficiency	0.835	0.050	21.28	<0.001			
	SIBER10	Impairment in real-life social engagements	0.806	0.051	20.24	<0.001			

Note. The first loading of each factor was fixed for model identification; therefore, SE, z, and *p* are not shown for those indicators. Item content is abbreviated/paraphrased; use the authorized scale wording in the instrument. α = Cronbach’s alpha; AVE = average variance extracted; CR = composite reliability; λ = standardized loading; — = not applicable.

**Table 4:** Correlations among cyberchondria, affect, and autonomic parameters (*N* = 600).

Variable	Mean ± SD	1	2	3	4	5	6	7	8	9	10
1. CSS-12 Total Score	35.46 ± 7.89	1									
2. Daily Screen Time (h)	5.24 ± 2.05	0.435 ***	1								
3. BMI (kg/m^2^)	22.86 ± 3.42	0.162 ***	0.126 **	1							
4. WHtR	0.45 ± 0.05	0.184 ***	0.142 **	0.748 ***	1						
5. DASS-21 Anxiety	6.84 ± 3.12	0.512 ***	0.348 ***	0.144 **	0.168 ***	1					
6. DASS-21 Stress	8.16 ± 3.45	0.486 ***	0.362 ***	0.138 **	0.154 ***	0.648 ***	1				
7. Resting HR (bpm)	74.6 ± 8.6	0.318 ***	0.284 ***	0.172 ***	0.198 ***	0.382 ***	0.341 ***	1			
8. MAP (mmHg)	90.7 ± 8.4	0.246 ***	0.198 ***	0.264 ***	0.294 ***	0.288 ***	0.274 ***	0.412 ***	1		
9. RMSSD (ms)	38.2 ± 11.4	−0.412 ***	−0.326 ***	−0.184 ***	−0.216 ***	−0.468 ***	−0.432 ***	−0.498 ***	−0.312 ***	1	
10. LF/HF ratio	1.82 ± 0.64	0.394 ***	0.310 ***	0.156 ***	0.182 ***	0.424 ***	0.398 ***	0.462 ***	0.286 ***	−0.614 ***	1

Note. Pearson correlations. CSS-12 = 12-item Cyberchondria Severity Scale; BMI = body mass index; WHtR = waist-to-height ratio; DASS-21 = Depression Anxiety Stress Scales-21; HR = heart rate; MAP = mean arterial pressure; RMSSD = root mean square of successive differences; LF/HF ratio = Low-frequency to high-frequency ratio. ** *p* < 0.01. *** *p* < 0.001.

**Table 5:** Autonomic and affective measures across exploratory CSS-12 score strata (*N* = 600).

CSS-12 Score Stratum	*n*	RMSSD (ms)	SDNN (ms)	LF/HF Ratio	Resting HR (bpm)	MAP (mmHg)	Anxiety Score
Low (<30)	148	44.8 ± 10.2	53.4 ± 12.1	1.48 ± 0.46	70.4 ± 7.2	87.8 ± 7.4	4.12 ± 2.04
Moderate (30–39)	296	38.6 ± 9.8	46.8 ± 11.8	1.76 ± 0.54	74.2 ± 8.0	90.4 ± 7.9	6.78 ± 2.62
High (≥40)	156	31.2 ± 9.1	39.6 ± 10.6	2.24 ± 0.68	80.1 ± 8.9	94.6 ± 8.8	9.84 ± 3.18
One-Way ANOVA (F)	—	58.42	44.18	61.26	48.74	28.91	142.60
*p*-value	—	<0.001	<0.001	<0.001	<0.001	<0.001	<0.001
Effect Size (ηp^2^)	—	0.164	0.129	0.170	0.140	0.088	0.323
Post Hoc (Tukey HSD)	—	Low > Mod > High	Low > Mod > High	High > Mod > Low	High > Mod > Low	High > Mod > Low	High > Mod > Low

Note. Values are mean ± SD unless otherwise indicated. Low = low score (CSS-12 < 40); Moderate = moderate score (CSS-12 30–39); High = high score (CSS-12 ≥ 40); RMSSD = root mean square of successive differences; SDNN = standard deviation of normal-to-normal intervals; LF/HF ratio = Low-frequency to high-frequency ratio; HR = heart rate; MAP = mean arterial pressure; — = not applicable. Score strata are exploratory and are not diagnostic categories. Effect size is partial ηp^2^.

**Table 6:** Hierarchical linear regression predicting RMSSD (*N* = 600).

Block/Predictor	B	SE	β	t	*p*	95% CI	VIF
Block 1: Basal & Anthropometric							
Constant	53.82	3.24	—	16.61	<0.001	[47.45, 60.19]	—
Age (years)	−0.32	0.16	−0.079	−2.00	0.046	[−0.63, −0.01]	1.036
Gender (Ref: Male)	−1.74	0.74	−0.094	−2.35	0.019	[−3.19, −0.29]	1.030
WHtR (Waist-to-height ratio)	−18.45	4.82	−0.158	−3.83	<0.001	[−27.92, −8.98]	1.054
Physical Inactivity (Ref: Active)	−3.38	0.76	−0.177	−4.45	<0.001	[−4.87, −1.89]	1.032
Model Summary: R^2^ = 0.091, Adjusted R^2^ = 0.085, F(4, 595) = 14.88, *p* < 0.001							
Block 2: Exposure & Affect							
Constant	59.40	3.36	—	17.68	<0.001	[52.80, 66.00]	—
Age (years)	−0.24	0.15	−0.059	−1.60	0.110	[−0.53, 0.05]	1.050
Gender	−1.18	0.68	−0.064	−1.74	0.083	[−2.51, 0.15]	1.042
WHtR	−13.20	4.46	−0.113	−2.96	0.003	[−21.96, −4.44]	1.068
Physical Inactivity	−2.42	0.70	−0.127	−3.46	<0.001	[−3.79, −1.05]	1.048
Daily Screen Time (hours)	−0.96	0.18	−0.172	−5.33	<0.001	[−1.31, −0.61]	1.186
DASS-21 Anxiety Score	−0.82	0.14	−0.225	−5.86	<0.001	[−1.09, −0.54]	1.348
Model Summary: ΔR^2^ = 0.141, ΔF(2, 593) = 54.62, *p* < 0.001, Cumulative R^2^ = 0.232							
Block 3: Cyberchondria Severity							
Constant	65.92	3.52	—	18.73	<0.001	[59.01, 72.83]	—
Age (years)	−0.16	0.14	−0.039	−1.14	0.254	[−0.44, 0.12]	1.056
Gender	−0.88	0.64	−0.048	−1.38	0.169	[−2.14, 0.38]	1.046
WHtR	−10.14	4.30	−0.087	−2.36	0.019	[−18.59, −1.69]	1.074
Physical Inactivity	−1.98	0.66	−0.104	−3.00	0.003	[−3.28, −0.68]	1.055
Daily Screen Time	−0.62	0.18	−0.111	−3.44	<0.001	[−0.97, −0.27]	1.268
DASS-21 Anxiety Score	−0.54	0.14	−0.148	−3.86	<0.001	[−0.81, −0.26]	1.492
CSS-12 Total Score	−0.37	0.06	−0.256	−6.17	<0.001	[−0.49, −0.25]	1.546
Model Summary: ΔR^2^ = 0.049, ΔF(1, 592) = 38.07, *p* < 0.001, Final R^2^ = 0.281, Adj. R^2^ = 0.272, D-W = 1.98							

Note. RMSSD is expressed in milliseconds; lower values indicate lower vagally mediated HRV. Gender reference = male; physical-activity reference = active. B = unstandardized coefficient; β = standardized coefficient; CI = confidence interval; VIF = variance inflation factor; D-W = Durbin–Watson statistic; WHtR = waist-to-height ratio; DASS-21 = Depression Anxiety Stress Scales-21; CSS-12 = 12-item Cyberchondria Severity Scale; — = not applicable.

## Data Availability

The de-identified data supporting the findings are available from the corresponding author upon reasonable request. The data are not publicly available because of participant privacy and the conditions of the ethics approval.

## Abbreviations

AVE, Average variance extracted; BF%, Body fat percentage; BMI, Body mass index; CFA, Confirmatory factor analysis; CFI, Comparative Fit Index; CI, Confidence interval; CR, Composite reliability; CSS-12, 12-item Cyberchondria Severity Scale; DASS-21, Depression Anxiety Stress Scales-21; HRV, Heart-rate variability; LF/HF, Low-frequency to high-frequency ratio; MAP, Mean arterial pressure; RMSEA, Root Mean Square Error of Approximation; RMSSD, Root mean square of successive differences; SDNN, Standard deviation of normal-to-normal intervals; SRMR, Standardized Root Mean Square Residual; TLI, Tucker–Lewis Index; VIF, Variance inflation factor; WHR, Waist-to-hip ratio; WHtR, Waist-to-height ratio.

## References

[B1-behavsci-16-01759] Agorastos A., Mansueto A. C., Hager T., Pappi E., Gardikioti A., Stiedl O. (2023). Heart rate variability as a translational dynamic biomarker of altered autonomic function in health and psychiatric disease. Biomedicines.

[B2-behavsci-16-01759] Arsenakis S., Chatton A., Penzenstadler L., Billieux J., Berle D., Starcevic V., Khazaal Y. (2021). Unveiling the relationships between cyberchondria and psychopathological symptoms. Journal of Psychiatric Research.

[B3-behavsci-16-01759] Bahadır O., Dündar C. (2024). The impact of online health information source preference on intolerance to uncertainty and cyberchondria in a youthful generation. Indian Journal of Psychiatry.

[B4-behavsci-16-01759] Baumgartner S. E., Hartmann T. (2011). The role of health anxiety in online health information search. Cyberpsychology, Behavior, and Social Networking.

[B5-behavsci-16-01759] Billman G. E. (2013). The LF/HF ratio does not accurately measure cardiac sympatho-vagal balance. Frontiers in Physiology.

[B6-behavsci-16-01759] Chaari I., Guidara H., Hadrich Z., Kessentini M., Abid W., Maaloul E., Kammoun A., Charfeddine F., Aribi L., Messedi N., Aloulou J. (2026). Cyberchondria and health anxiety among Tunisian university students: A cross-sectional study. European Psychiatry.

[B7-behavsci-16-01759] Chalmers J. A., Quintana D. S., Abbott M. J.-A., Kemp A. H. (2014). Anxiety disorders are associated with reduced heart rate variability: A meta-analysis. Frontiers in Psychiatry.

[B8-behavsci-16-01759] Choi H., Jeong G. (2021). Characteristics of the measurement tools for assessing health information-seeking behaviors in nationally representative surveys: Systematic review. Journal of Medical Internet Research.

[B9-behavsci-16-01759] Chu J. T. W., Wang M. P., Shen C., Viswanath K., Lam T. H., Chan S. S. C. (2017). How, when, and why people seek health information online: Qualitative study in Hong Kong. Interactive Journal of Medical Research.

[B10-behavsci-16-01759] Ciułkowicz M., Misiak B., Szcześniak D., Luc D., Wieczorek T., Fila-Witecka K., Gawłowski P., Rymaszewska J. (2022). Social support mediates the association between health anxiety and quality of life: Findings from a cross-sectional study. International Journal of Environmental Research and Public Health.

[B11-behavsci-16-01759] Dadaczynski K., Okan O., Messer M., Leung A. Y. M., Rosário R., Darlington E., Rathmann K. (2021). Digital health literacy and web-based information-seeking behaviors of university students in Germany during the COVID-19 pandemic: Cross-sectional survey study. Journal of Medical Internet Research.

[B12-behavsci-16-01759] Esparza-Ros F., Vaquero-Cristóbal R., Marfell-Jones M. (2019). International standards for anthropometric assessment.

[B13-behavsci-16-01759] Hsu W.-C. (2024). Developing a cyberchondria severity scale to promote self-care among university students during COVID-19. Scientific Reports.

[B14-behavsci-16-01759] Hsu W.-C., Chen P.-H., Hsu Y.-P. (2021). Undergraduate students’ online health information-seeking behavior during the COVID-19 pandemic. International Journal of Environmental Research and Public Health.

[B15-behavsci-16-01759] Keskiner E. Ş., Şahin E., Topkaya N., Yiğit Z. (2025). Behavioral emotion regulation strategies and symptoms of psychological distress among Turkish university students. Behavioral Sciences.

[B16-behavsci-16-01759] Laborde S., Mosley E., Thayer J. F. (2017). Heart rate variability and cardiac vagal tone in psychophysiological research—Recommendations for experiment planning, data analysis, and data reporting. Frontiers in Psychology.

[B17-behavsci-16-01759] Lagoe C., Atkin D. (2015). Health anxiety in the digital age: An exploration of psychological determinants of online health information seeking. Computers in Human Behavior.

[B18-behavsci-16-01759] Lovibond S. H., Lovibond P. F. (1995). Manual for the depression anxiety stress scales.

[B19-behavsci-16-01759] Luo A., Qin L., Yuan Y., Yang Z., Liu F., Huang P., Xie W. (2022). The effect of online health information seeking on physician–patient relationships: Systematic review. Journal of Medical Internet Research.

[B20-behavsci-16-01759] Mathes B. M., Norr A. M., Allan N. P., Albanese B. J., Schmidt N. B. (2018). Cyberchondria: Overlap with health anxiety and unique relations with impairment, quality of life, and service utilization. Psychiatry Research.

[B22-behavsci-16-01759] McElroy E., Kearney M., Touhey J., Evans J., Cooke Y., Shevlin M. (2019). The CSS-12: Development and validation of a short-form version of the cyberchondria severity scale. Cyberpsychology, Behavior, and Social Networking.

[B21-behavsci-16-01759] McElroy E., Shevlin M. (2014). The development and initial validation of the Cyberchondria Severity Scale (CSS). Journal of Anxiety Disorders.

[B23-behavsci-16-01759] McMullan R. D., Berle D., Arnáez S., Starcevic V. (2019). The relationships between health anxiety, online health information seeking, and cyberchondria: Systematic review and meta-analysis. Journal of Affective Disorders.

[B24-behavsci-16-01759] Mecherques-Carini M., Albaladejo-Saura M., Vaquero-Cristóbal R., Baglietto N., Esparza-Ros F. (2024). Validity and agreement between dual-energy X-ray absorptiometry, anthropometry and bioelectrical impedance in the estimation of fat mass in young adults. Frontiers in Nutrition.

[B25-behavsci-16-01759] Mestre-Bach G., Potenza M. N. (2023). Cyberchondria: A growing concern during the COVID-19 pandemic and a possible addictive disorder?. Current Addiction Reports.

[B26-behavsci-16-01759] Muse K., McManus F., Leung C., Meghreblian B., Williams J. M. G. (2012). Cyberchondriasis: Fact or fiction? A preliminary examination of the relationship between health anxiety and searching for health information on the Internet. Journal of Anxiety Disorders.

[B27-behavsci-16-01759] Santoro G., Starcevic V., Scalone A., Cavallo J., Musetti A., Schimmenti A. (2022). The doctor is in(ternet): The mediating role of health anxiety in the relationship between somatic symptoms and cyberchondria. Journal of Personalized Medicine.

[B28-behavsci-16-01759] Sarıçam H. (2018). The psychometric properties of Turkish version of Depression Anxiety Stress Scale-21 (DASS-21) in health control and clinical samples. Journal of Cognitive Behavioral Psychotherapies and Research.

[B29-behavsci-16-01759] Schaffarczyk M., Rogers B., Reer R., Gronwald T. (2022). Validity of the polar H10 sensor for heart rate variability analysis during resting state and incremental exercise in recreational men and women. Sensors.

[B30-behavsci-16-01759] Schenkel S. K., Jungmann S. M., Gropalis M., Witthöft M. (2021). Conceptualizations of cyberchondria and relations to the anxiety spectrum: Systematic review and meta-analysis. Journal of Medical Internet Research.

[B31-behavsci-16-01759] Sharot T., Sunstein C. R. (2020). How people decide what they want to know. Nature Human Behaviour.

[B32-behavsci-16-01759] Singh K., Brown R. J. (2014). Health-related Internet habits and health anxiety in university students. Anxiety, Stress, & Coping.

[B33-behavsci-16-01759] Starcevic V., Berle D. (2013). Cyberchondria: Towards a better understanding of excessive health-related Internet use. Expert Review of Neurotherapeutics.

[B34-behavsci-16-01759] Task Force of the European Society of Cardiology and the North American Society of Pacing and Electrophysiology (1996). Heart rate variability: Standards of measurement, physiological interpretation, and clinical use. Circulation.

[B35-behavsci-16-01759] te Poel F., Baumgartner S. E., Hartmann T., Tanis M. (2016). The curious case of cyberchondria: A longitudinal study on the reciprocal relationship between health anxiety and online health information seeking. Journal of Anxiety Disorders.

[B36-behavsci-16-01759] Thayer J. F., Åhs F., Fredrikson M., Sollers J. J., Wager T. D. (2012). A meta-analysis of heart rate variability and neuroimaging studies: Implications for heart rate variability as a marker of stress and health. Neuroscience & Biobehavioral Reviews.

[B37-behavsci-16-01759] Tuğtekin U., Barut Tuğtekin E. (2021). Siberkondri ciddiyet Ölçeği’nin kısa formunun Türkçeye uyarlanması ve öğretmen adaylarının aşırı çevrim içi bilgi arama davranışları. Anemon Muş Alparslan Üniversitesi Sosyal Bilimler Dergisi.

[B38-behavsci-16-01759] Varer Akpinar C., Mandiracioglu A., Ozvurmaz S., Kurt F., Koc N. (2023). Cyberchondria and COVID-19 anxiety and internet addiction among nursing students. Current Psychology.

[B39-behavsci-16-01759] Vismara M., Vitella D., Biolcati R., Ambrosini F., Pirola V., Dell’Osso B., Fineberg N. A. (2021). The impact of COVID-19 pandemic on searching for health-related information and cyberchondria on the general population in Italy. Frontiers in Psychiatry.

[B40-behavsci-16-01759] Yang C., Xu R. H. (2025). Impact of cyberchondria on health and quality of life: Scoping review. Journal of Medical Internet Research.

[B41-behavsci-16-01759] Yao Z., Qin N., Shi S., Li X., Zhong Z. (2026). Cyberchondria among college students and associated factors: A latent profile analysis. Scientific Reports.

[B42-behavsci-16-01759] Zhu X., Zheng T., Ding L., Zhang X. (2023). Exploring associations between eHealth literacy, cyberchondria, online health information seeking and sleep quality among university students: A cross-sectional study. Heliyon.

